# In Sync: The Effect of Physiology Feedback on the Match between Heart Rate and Self-Reported Stress

**DOI:** 10.1155/2015/134606

**Published:** 2015-06-04

**Authors:** Elisabeth T. van Dijk, Joyce H. D. M. Westerink, Femke Beute, Wijnand A. IJsselsteijn

**Affiliations:** Eindhoven University of Technology, P.O. Box 513, 5600 MB Eindhoven, Netherlands

## Abstract

Over the past years self-tracking of physiological parameters has become increasingly common: more and more people are keeping track of aspects of their physiological state (e.g., heart rate, blood sugar, and blood pressure). To shed light on the possible effects of self-tracking of physiology, a study was conducted to test whether physiology feedback has acute effects on self-reported stress and the extent to which self-reported stress corresponds to physiological stress. In this study, participants executed several short tasks, while they were either shown visual feedback about their heart rate or not. Results show that self-reported stress is more in sync with heart rate for participants who received physiology feedback. Interactions between two personality factors (neuroticism and anxiety sensitivity) and feedback on the level of self-reported stress were found, indicating that while physiology feedback may be beneficial for individuals high in neuroticism, it may be detrimental for those high in anxiety sensitivity. Additional work is needed to establish how the results of this study may extend beyond immediate effects in a controlled lab setting, but our results do provide a first indication of how self-tracking of physiology may lead to better body awareness and how personality characteristics can help us predict which individuals are most likely to benefit from self-tracking of physiology.

## 1. Introduction

Recent developments in mobile sensors and wearable devices allow for ubiquitous 24/7 tracking of individual activity and physiological states. Although these technologies are already well-known in specific application areas of mobile health (see, for example [1, 2]) and (semi-)professional sports (see, for example, [3, 4]), the worldwide growth in the use of smartphones and tablets has accelerated these developments. Data can now be generated on a broad range of physiological and behavioral indices, including a person's movement, location, social behavior, and even tone of voice. Applications are being introduced that prompt us to record data about our moods, sleep patterns, menstrual cycles, diet, exercise habits, stress levels, and so on. But is all this self-tracking and feedback really helpful, or healthy? A record of day-to-day variation in physiology may provide important health parameters for people as they manage a chronic illness or as indicators to a healthy person about their well-being. At the same time though, we need to consider the possibility that continuous health feedback may feed health anxiety, and thereby stress, rather than ameliorate it. Through continuous health data entry as well as health feedback, an excessive self-focus and preoccupation with one's health may be induced or strengthened. Thus, paradoxically, technology-based body awareness may become a catalyst of rumination and worry about one's health, a self-tracking version of the so-called “white coat syndrome.” The extent to which such an effect may or may not occur is an empirical question that, to date, has not been sufficiently addressed. The current paper aims to address this topic by experimentally investigating the relationship between continuous physiology feedback (i.e., heart rate information) and self-reported stress levels. In addition to gauging immediate effects of physiology feedback, potential moderating effects of personality variables are also explored.

Many existing mind-body practices involve a component of body awareness [5]. These mind-body practices are believed to have positive effects; in a review, Astin, Shapiro, Eisenberg, and Forys conclude that there is considerable evidence for the positive effects of several mind-body practices on diseases and symptoms ranging from headaches and insomnia to coronary artery disease [6]. The kind of body awareness practiced in these interventions, however, is different from the awareness created in self-tracking in two ways. First, these practices specifically teach a nonjudgmental awareness, where sensations are observed without appraisal, analysis, or judgment [5]. This is not typically the case in self-tracking of physiology. In fact, analysis and interpretation of data is often encouraged in self-tracking systems. Second, the body awareness promoted by these practices is based on interoception and proprioception: perception of internal processes (e.g., heart beats) and body positioning by means of internal cues. This is in line with the idea that these interventions seek to increase a sense of “embodiment” [5], the natural integration of the body as part of the self. The body awareness created in self-tracking of physiology is more indirect, as it is achieved by externalizing objective measurements of physiological signals. This seems to be opposed to the idea of embodiment, focusing rather on the dichotomy between body and self than on the unison of the two.

Biofeedback is a special case in the world of mind-body medicine when it comes to body awareness. Biofeedback, including biofeedback-assisted relaxation, has been found to be efficacious in treating problems ranging from headaches, anxiety, and motion sickness to attention deficit hyperactivity disorder (ADHD) and epilepsy (see [7] for a review). As is the case in self-tracking of physiology, biofeedback promotes awareness of internal signals by measuring them objectively and then presenting them via some external channel. There is, however, a crucial difference between self-tracking of physiology and biofeedback in terms of body awareness. In biofeedback, body awareness is not a goal in itself, but rather a stepping stone, which helps people learn to voluntarily control and improve the physiological parameters being measured. In line with this, the feedback about the person's physiological state during the training sessions is constant and immediate, so any changes in physiology are immediately observable. This helps the person learn to deliberately affect the physiological process in question. In contrast, this training in control over physiological processes is generally not included in self-tracking systems.

As things stand, it is unclear what the effects of exposure to one's own physiological measurements are. There is evidence that other interventions that promote body awareness have positive effects, but there are important differences between those interventions and self-tracking of physiology that make it difficult to determine whether the positive effects found will generalize to self-tracking of physiology. As a first step toward investigating the effects of self-tracking, we focus on the acute effects of physiology feedback. We have therefore performed a study where some participants were given feedback about their heart rate while performing several small tasks, while other participants performed the same tasks, but they were not given physiology feedback.

We hypothesize that participants may (consciously or otherwise) use physiology feedback to inform (reports about) their subjective experience of the tasks. To investigate this possibility, we collected momentary self-reported stress levels after each task. Stress was chosen here for several reasons: firstly, there is an intuitive connection between stress and heart rate. Secondly, (long-term) stress is an important factor in psychosomatic illness and is believed to be the cause of many health complaints. As such, stress is seen as an aspect of wellness that is of significant societal importance, and the significance of stress is intuitively understood by most.

In addition to momentary reports of stress, retrospective reports of the same are of interest here since retrospective self-report normally tends to deviate from momentary self-report, the latter often being more closely related to physiological measures [8]. If physiology feedback makes participants more aware of their stress level in the moment, it may also help them to remember and compare those stress experiences in hindsight, bringing retrospective self-report closer to momentary self-report and physiological measurements.

Finally, we hypothesize that the effects of physiology feedback may not be the same for all individuals but may be moderated by certain personality variables. Two personality variables were selected as possible moderators. The first is neuroticism, which represents emotional instability, or the tendency to experience negative emotions [9]. As those high in neuroticism believe that they are easily stressed, this expectation may moderate the effect of physiology feedback, especially if the feedback relates to stress. The second personality variable included in this study is anxiety sensitivity, which reflects the tendency to experience anxiety or fear in response to (benign) body sensations associated with anxiety and believing these sensations to be harmful or a reflection of serious illness [10]. If physiology feedback about stress is either seen as a sign of anxiety or results in a heightened body awareness, those high in anxiety sensitivity may show heightened levels of anxiety in response to physiology feedback about stress.

## 2. Method

Besides the actual content of the feedback, the interpretation of that information may also play a role in the effect of the feedback. Some terms or parameters may be easier for participants to interpret. For instance, the significance of a high stress score is probably more easily understood by most than a low heart rate variability (even though this is also considered an indicator of stress [11]). In addition the physiological measurement itself may affect people. Medical settings and measurements are known to cause anxiety in many, as illustrated by phenomena like white-coat hypertension (i.e., over 20% of people show increased blood pressure when measured in a doctor's office [12]). To tease these different aspects of physiology feedback apart, different levels of feedback were used.

### 2.1. Design

A 3 by 20 mixed design was used, comprising 20 different tasks as a within-subjects factor and 3 feedback types as a between-subjects factor.

Participants performed 20 brief (1 minute) tasks. For descriptions of the tasks, see Table 1. Half the tasks were designed to be mildly stressful, the other half more relaxing. To avoid a build-up of stress as a result of having many stressful tasks in a row, stressful tasks were alternated with relaxing ones. To counterbalance the order of the tasks as well as possible within this scheme, a set of 10th order digram-balanced graecolatin squares were used [13], resulting in a design where (a) all stressful tasks appear in every position equally often, (b) all relaxing tasks appear in every position equally often, (c) every possible order of two consecutive stressful tasks occurs equally often, (d) every possible order of two relaxing tasks occurs equally often, and (e) every combination of stressful and relaxing tasks occurs equally often.

To better understand what aspect of physiology feedback affects participants, we divided our participants into three groups: a “stress-feedback” group, who were told the physiology feedback reflected their stress level; a “HR-feedback” group, who were told the physiology feedback reflected their heart rate; and a “measurement-only” group, who wore ECG devices, but did not get physiology feedback.

### 2.2. Participants

The experiment was conducted in accordance with the Declaration of Helsinki (1964). All participants were informed in full about the experiment prior to participation and gave their informed consent. Participants were allowed to withdraw at any time during the experiment, without any adverse consequences. The experiment was approved by the local ethics committee at Eindhoven University of Technology, Human-Technology Interaction Group.

A total of 74 participants participated in the experiment (38 male, 36 female). The age of participants ranged from 18 to 67 years (average 27 years). Participants were recruited from a local participant database. People with a history of cardiac disease and/or mental illness were excluded from participation, by noting this point in both the invitation to participate and the informed consent. Participants were instructed beforehand not to smoke, engage in rigorous exercise, or drink caffeinated or alcoholic beverages in the 2 hours preceding the experiment. At the end of the experiment they were asked if they had indeed refrained from doing these things.

### 2.3. Stimuli

Descriptions of the tasks used in the experiment can be found in Table 1. The tasks were pretested to ensure they evoke a wide enough range in self-reported stress and average heart rate.

**Figure 1:**
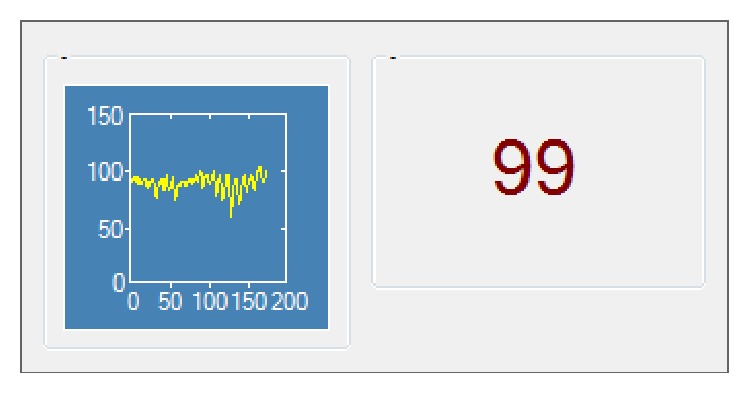
Screenshot of the physiology feedback window.

For the physiology feedback, a custom application was used consisting of two parts (see Figure 1). On the left, a graph was shown that displayed the participant's heart rate over approximately the last 3 minutes; the horizontal axis of the graph was automatically readjusted when the graph approached the end of the currently shown time window. On the right, the current heart rate was shown in beats per minute, calculated as a moving average over the last 20 beats to avoid rapid fluctuations in the signal. The physiology feedback window was shown in the top-right corner of the screen and did not overlap with the software used to present the tasks.

### 2.4. Measurements

Both physiological data and subjective data were gathered during the experiment.

#### 2.4.1. Physiological Measurements

For collection of ECG data, three Kendall H124SG ECG electrodes were used in the standard lead-II placement: the ground on top of the collar bone near the left shoulder, one electrode under the collar bone near the right shoulder, and one electrode underneath the ribs on the left side of the torso. A sampling frequency of 1024 Hz was used. From the raw ECG data, RR intervals were extracted and the average heart rate was subsequently calculated for each task.

#### 2.4.2. Subjective Experience

After each task, subjects responded to four custom items about their experience of that task (see Table 2): “I felt (stressed, calm, relaxed, and tense) during this task.” A 7-point Likert scale ranging from “disagree completely” (1) to “agree completely” (7) was used for each item. A factor analysis revealed only one underlying factor (note that items 2 and 3 were reverse-coded). This seems to warrant the use of these questions as a single scale. A reliability test showed that Cronbach's Alpha for this scale is high (*α* = 0.949). For the remainder of the analyses we therefore use the average (again, with items 2 and 3 reverse-coded) of these four items as a single momentary stress score.

**Figure 2:**
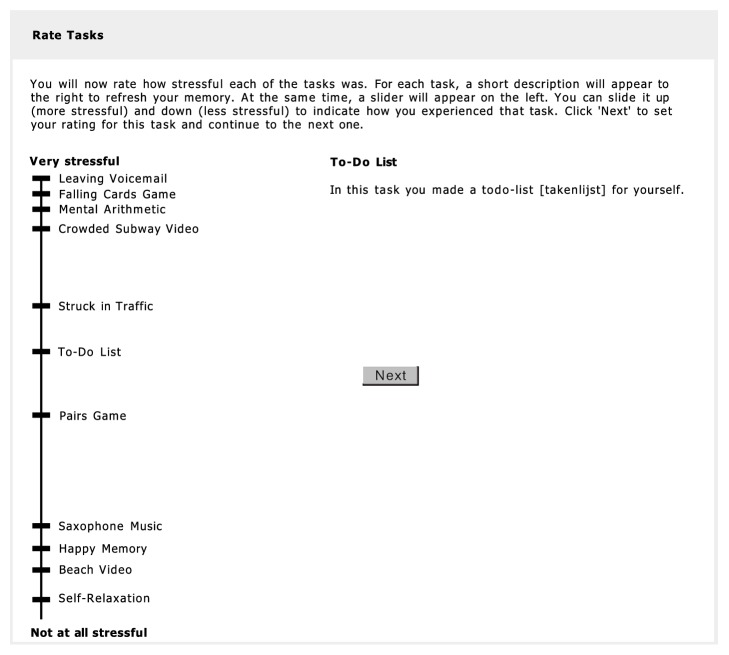
Screenshot of the task stress rating, which was administered after all tasks were completed. Each task was presented with a brief description. The participant could then place the task on the scale by sliding the relevant marker up or down. Once the participant proceeded to placement of the next task, placement of the previous task was fixed and could no longer be altered.

After subjects completed all tasks, they again rated all tasks on stressfulness, but this time in a retrospective and comparative way. The scale here ranged from 0 (“not at all stressful”) to 100 (“very stressful”) and participants added the tasks to the scale one by one, while the placement of earlier tasks remained visible to facilitate comparison of the tasks and consistent use of the scale. See Figure 2 for a screenshot of this rating task.

#### 2.4.3. Personality Factors

After the tasks and rating task were completed, participants filled out two personality-related questionnaires: firstly, the 8 items on neuroticism from the Big Five Personality Inventory [9], reflecting emotional instability, or the tendency to experience negative emotions, and secondly the 16-item Anxiety Sensitivity Index [10], reflecting the tendency to experience anxiety or fear in response to (benign) body sensations associated with anxiety and believing these sensations to be harmful or a reflection of serious illness. These scales were included as possible moderators of effects of physiology feedback.

Overall scores for neuroticism and ASI were calculated for each participant, as the average of individual item scores of the relevant questionnaire (with reverse coding for the appropriate items in the neuroticism questionnaire). The neuroticism questionnaire showed a high reliability in our sample (Cronbach's *α* = 0.807), as did the Anxiety Sensitivity Index (Cronbach's *α* = 0.841).

#### 2.4.4. Manipulation Check

To assess whether the feedback type manipulation had worked, participants who received physiology feedback during the experiment were asked at the end of the experiment how often they had looked at the feedback, on a scale from 1 (“not at all”) to 7 (“very much”).

### 2.5. Procedure

When participants came into the lab, an informed consent was administered. After signing the informed consent, participants first performed a practice task at a computer, where they were instructed to relax as much as possible, while keeping their eyes open (the same as task 11 from the main experiment). The practice task was followed by the same four stress self-report items as the tasks in the main experiment.

Participants were then asked to apply the necessary electrodes for the ECG measurement. The ECG recording software was started, and for the HR-feedback and stress-feedback groups the feedback application was started as well. The HR-feedback group were told the feedback application would show their heart rate, while the stress-feedback group were told the application would show their stress level.

The 20 tasks were then presented to all participants via a computer program, each followed by the stress self-report items. After all 20 tasks were completed, participants filled out the postquestionnaires (task stressfulness ratings, neuroticism and anxiety sensitivity scales, and manipulation check). Finally, all participants were paid and thanked for their participation.

## 3. Results

In our analyses, we tested the effects of feedback type on several dependent variables. Firstly, does feedback type affect the self-reported level of momentary and retrospective stress? Secondly, does feedback type affect the extent to which momentary and retrospective stress self-report are aligned? And thirdly, does feedback type affect the extent to which (momentary or retrospective) self-reported stress is in line with heart rate?

The last two dependent variables are both related to a “match” between two different measures of stress. We have devised two different measures to operationalize this concept of a match between two variables. The first measure is calculated on the individual level and consists of taking, for each participant, the correlation between the two relevant variables (i.e., momentary and retrospective stress, or heart rate and momentary stress, or heart rate and retrospective stress). An intrapersonal correlation is used here, as individual differences may exist in both baseline and variability on the different measures of stress.

The second measure is calculated on the task level. To this end we converted the raw heart rate and stress scores to a more comparable form by transforming them into *z*-scores, using within-person averages and standard deviations of the relevant variable. The absolute differences between the heart rate *z*-scores and stress *z*-scores, as well as between momentary and retrospective stress *z*-scores, were then calculated for each task, for each participant. These difference scores are a lower-is-better representation of the match between the two relevant variables.

### 3.1. Outliers

Data from a total of 8 participants had to be excluded for a number of reasons. For two participants, the ECG data could not be preprocessed, likely due to an electrode being faulty or not properly connected. One participant seemed to have misunderstood the intended use of the retrospective stressfulness scale, simply leaving the markers for all tasks at the place they were initially presented. One participant showed an exceptionally low (>4 standard deviations below the mean) correlation between momentary and retrospective stress (a parameter that is to be used in one of our analyses). Finally, 4 additional participants reported having exercised or consumed caffeine before the experiment. All data from these 8 participants was excluded from further analysis. This brought the total number of participants in the data set used for analysis to 66.

### 3.2. Manipulation Check

Results from the manipulation check show that the manipulation was successful: on average, participants in the feedback groups reported that they had looked at the physiology feedback quite often (an average of 5.27 on a scale from 1, not at all, to 7, very often). No one claimed never to have looked at the feedback.

### 3.3. Effects of Feedback Type on Momentary Stress and Retrospective Stress

To analyze the effect of feedback type on the levels of momentary and retrospective self-reported stress, a series of multilevel models was used. The data we have is hierarchical in nature: there are 3 feedback type groups, each containing several participants, each of whom performed 20 tasks. Multilevel models are aimed at modeling these different levels of the hierarchy. Separate models were estimated for momentary stress and retrospective stress. The initial model included “feedback type” as a fixed factor and “task” as a repeated measure. This model showed no significant main effect of feedback type on momentary stress for either momentary (*F*(2,587) = 1.575, *P* = ns) or retrospective stress (*F*(2,840) = 0.194, *P* = ns).

In a second iteration, two interaction terms (“neuroticism ∗ feedback type” and “ASI ∗ feedback type”) were added to the model to test the moderating effects of the personality factors. This addition significantly improved the fit of the overall model, as indicated by the change in −2 Log Likelihood (*χ* ^2^(6) = 21.786, *P* < 0.005 for momentary stress, *χ* ^2^(6) = 18.346, *P* < 0.01 for retrospective stress). For both momentary and retrospective stress, the parameter estimate (conceptually similar to a regression coefficient, see Tables 3 and 4) for the “neuroticism ∗ feedback type” interaction was positive and significant specifically in the measurement-only group, indicating that in this group, but not the other groups, participants with a higher level of neuroticism generally reported higher levels of momentary (*t*(583) = 2.868, *P* = 0.004) and retrospective (*t*(1086) = 2.050, *P* = 0.041) stress. The parameter estimate for the “ASI ∗ feedback type” interaction was positive and significant specifically in the stress-feedback group, indicating that in this group, but not the other groups, participants who score higher on ASI generally reported higher levels of momentary (*t*(583) = 2.172, *P* = 0.030) and retrospective (*t*(1086) = 2.219, *P* = 0.027) stress.

### 3.4. Match between Heart Rate, Momentary Stress, and Retrospective Stress

**Figure 3:**
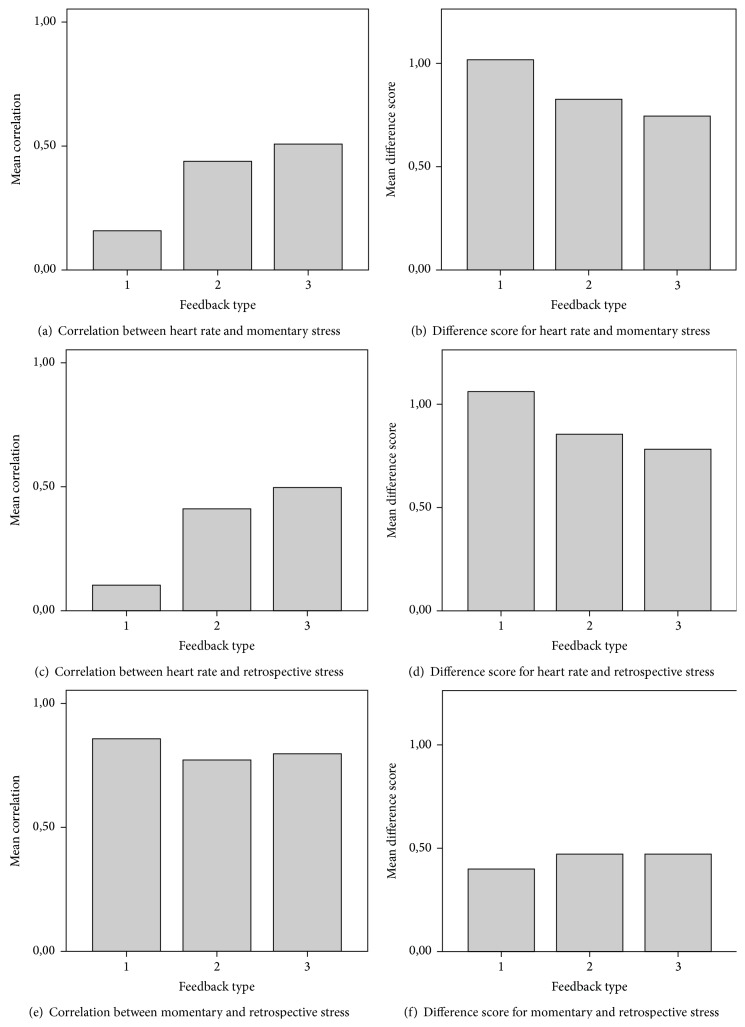
Average correlations (left) and difference scores (right) between different variables for the different feedback types. A higher correlation means a stronger match between two variables, while the reverse is true for the difference scores. For the sake of readability, feedback types are coded as 1 = measurement-only, 2 = HR-feedback, and 3 = stress-feedback.

To test how feedback type affects the extent to which participants' subjective stress levels are in line with their heart rate and how well participants remember their self-reported stress levels, a MANOVA was used. In this analysis, feedback type was the independent variable and the correlation coefficients between each combination of two stress measures were dependent variables (see the beginning of Section 2 and Figure 3).

The analysis showed that the average intrapersonal correlation between heart rate and both momentary and retrospective stress was significantly different for different feedback types (*F*(2,63) = 12.074, *P* < 0.001 for momentary stress, *F*(2,63) = 16.424, *P* < 0.001 for retrospective stress). A series of LSD post-hoc tests revealed that, for both momentary and retrospective stress, the correlation with heart rate was significantly higher in the HR-feedback group than the measurement-only group (*P* = 0.001 for momentary stress, *P* < 0.001 for retrospective stress), but there was no significant difference between the stress-feedback and the HR-feedback groups in terms of the correlation with heart rate. The analysis showed no significant effect of feedback type on the correlation between momentary and retrospective stress (*F*(2,63) = 2.309, *P* = ns).

The downside of the analysis described above is that, by collapsing the data into correlation coefficients per person, an aspect of the data is lost: the variability between tasks. As mentioned before, multilevel models can be used to model the different levels in our hierarchical data. For these multilevel models, a measure similar to the correlations used above is needed that can be calculated at the level of individual tasks for each person. The difference scores described in the beginning of Section 3 were used for this purpose.

To reestablish the significant results obtained in the MANOVA for the correlations between both momentary and retrospective stress and heart rate, a simple model ignoring the hierarchical nature of the data was first tested. This model included only “feedback type” as a fixed factor. Separate analyses were performed for the HR versus momentary stress difference score and the HR versus retrospective stress difference score. As expected, the “feedback type” factor had a highly significant effect on both the HR versus momentary stress difference score (*F*(2,1320) = 19.742, *P* < 0.001) and the HR versus retrospective stress difference score (*F*(2,1320) = 21.360, *P* < 0.001).

In a second iteration, “task” was added to the model as a repeated measure. This addition significantly improved the fit of the overall model, as indicated by the change in −2 Log Likelihood (*χ* ^2^(19) = 67.744, *P* < 0.001 for HR versus momentary stress, *χ* ^2^(19) = 67.744, *P* < 0.001 for HR versus retrospective stress). The “feedback type” factor was still significant for both dependent variables: *F*(2,1204) = 17.342, *P* < 0.001 for HR versus momentary stress, with a significant contrast between the measurement-only and HR-feedback groups (*P* < 0.001) and a marginally significant contrast between the HR-feedback and stress-feedback groups (*P* = 0.057); and *F*(2,1234) = 20.105, *P* < 0.001 for HR versus retrospective stress, with a significant contrast between the measurement-only and HR-feedback groups (*P* < 0.001) and a marginally significant contrast between the HR-feedback and stress-feedback groups (*P* = 0.063).

In a third iteration, the personality factors were included in the model by adding two interaction terms (“neuroticism ∗ feedback type” and “ASI ∗ feedback type”). The fit of the model was not significantly improved by these additions (*χ* ^2^(6) = 6.961, *P* = ns for HR versus momentary stress, *χ* ^2^(6) = 7.589, *P* = ns for HR versus retrospective stress), indicating that the effect of the feedback was not significantly different for different scores on the personality factors.

## 4. Discussion

The goal of this study was to assess some of the acute effects of physiology feedback, to better understand the effects self-tracking of physiology might have. Specifically, we investigated to what extent physiology feedback would affect the match between participants' physiological stress and their self-reported stress. In addition, the effect of physiology feedback on participants' memory for self-reported stress (i.e., the match between momentary and retrospective stress) was investigated. Finally, we examined whether physiology feedback might affect the self-reported stress level itself and explored the role of two personality variables (neuroticism and anxiety sensitivity) in this respect.

Our results show that when participants are given feedback about their heart rate, their estimates of their stress level become more in tune with their heart rate: when their heart rate is high, they report a high stress level, and when their heart rate is low, they report a low stress level. The effect seems to be stronger in the stress-feedback group compared to the HR-feedback group (although the effect was only marginally significant), suggesting that the instructions given about how to interpret the feedback might be important.

There are at least two explanations for the fact that physiology feedback resulted in a better match between stress self-report and heart rate. The first is that the physiology feedback helps people to become more aware of their body and that this heightened awareness then informs their subjective stress reports. Alternatively, people may see the feedback as a more objective and more accurate source of information about their stress level than their own experience. This might mean that when their subjective experience does not match the feedback, they use the feedback rather than their own experience to inform their reports about stress. The current study cannot provide conclusive evidence either way, but a follow-up study using false heart rate feedback might: if the second explanation is true, false heart rate feedback should cause self-report to become more in tune with the false feedback; if the first explanation is true, false heart rate feedback either might have no effect (if false feedback is not effective at triggering body awareness) or might cause stress self-report to become more in tune with one's own heart rate.

Physiology feedback was found not to affect the extent to which retrospective self-reported stress matches momentary self-reported stress. This implies that although physiology feedback made subjective reports of stress more in tune with heart rate, it did not help participants to better remember their stress levels. Although this finding may well represent a true effect, it may also have been caused by a ceiling effect: the correlation between momentary and retrospective stress self-report was already quite high (*r* = 0.8 on average) for almost all participants in the measurement-only group, leaving little room for improvement. This may have been due to the limited time interval between the momentary and retrospective reports: even for the first task, the momentary and retrospective assessment were typically no more than 25 minutes apart. A longer interval might give rise to different results as a consequence of temporal decay of short-term memory, as well as other effects that could influence delayed recall.

Our results did not show an effect of physiology feedback on self-reported stress, indicating that, on average, physiology feedback did not cause participants to become more or less stressed. The interaction between neuroticism and feedback on self-reported stress levels, however, was significant. Specifically, it was found that, in the measurement-only condition, a higher score on neuroticism predicted higher stress levels, while this effect was not present in the feedback conditions. Most items in the neuroticism questionnaire are related to whether participants see themselves as tense, nervous, or easily upset. Those who score high on this scale presumably believe they experience relatively high levels of stress. This might explain why those who score high on neuroticism report higher stress levels than low-neuroticism individuals in the measurement-only condition. For individuals with a high score on neuroticism, the physiology feedback may provide good news, in the sense that their objective, physiological stress response may be less strong than they expected. This might explain why the difference in self-reported stress between higher- and lower-neuroticism individuals disappears in the HR-feedback and stress-feedback conditions.

The interaction between anxiety sensitivity and feedback on self-reported stress was also found to be significant. In the stress-feedback group, individuals who scored higher on anxiety sensitivity were shown to report higher levels of stress. Individuals with a high level of anxiety sensitivity tend to experience anxiety when they detect anxiety-related body sensations [10]. For these individuals, physiology feedback might draw their attention to benign physiological responses, thereby causing them to become more anxious and report higher levels of stress. This may explain our results and suggests that self-tracking of physiology might have adverse effects for individuals with a high level of anxiety sensitivity. Note that the effect was only found in the stress-feedback and not in the HR-feedback condition, suggesting again that the explanation given about the meaning of the feedback parameter may be important in certain contexts.

On a general note, there are several differences between our lab setting and self-tracking in the field that limit the generalizability of our results: firstly, the pervasive feedback used in this study is different from the physiology feedback obtained in self-tracking, which is generally available on demand, rather than being presented constantly. Secondly, self-tracking of physiology involves other interactions with one's data besides the real-time physiology feedback used in this study (e.g., feedback after the fact, viewing data aggregated over longer periods of time). Finally, this study only assessed acute effects of physiology feedback, precluding any conclusions about longer-term effects. Still, even though this study does not yet paint a complete picture of the effects of self-tracking of physiology, it does provide insight into how self-tracking of physiology may lead to better body awareness and how individual differences in personality characteristics may moderate the effects of self-tracking on stress.

## 5. Conclusion

A study was conducted to test whether physiology feedback has acute effects on self-reported stress and the extent to which it is in sync with physiological stress. In this study, participants executed several short tasks, while they were either shown visual feedback about their current and past heart rate or not. The group who received feedback was further subdivided into a group who were told the feedback represented their heart rate and a group who were told the feedback represented their stress level.

Results show that self-reported stress is more in sync with heart rate for participants who received physiology feedback. This implies that either the feedback helps these participants to better detect their own physiological stress or they use the external feedback to inform their reports of experienced stress. Either way, these results suggest that physiology feedback increases body awareness on a subjective—if short-term—level.

Physiology feedback was found not to affect participants' stress levels. However, interactions between two personality factors and feedback on stress levels were found. Firstly, participants with a high score on neuroticism were found to report more stress than those with a low score on neuroticism when no physiology feedback was given, but this difference disappeared when physiology feedback was provided. This may reflect a belief-based bias, as neuroticism reflects the degree to which people believe they are prone to episodes of stress, tension, and upset. This in turn suggests that physiology feedback may be effective at reducing self-reported stress when it gives “good news.” Secondly, individuals high in anxiety sensitivity were found to report higher levels of stress in the condition where physiology feedback about stress was given, suggesting that self-tracking of physiology may be less suitable for these individuals.

The results found in this study are based on experiences in a lab setting which, although well-controlled, lacks the longitudinal nature and richness of interaction of real-world self-tracking. How the results of this study translate to long-term effects of self-tracking on general health and well-being is therefore a matter that will require additional work. Nevertheless, our findings give a first indication of how self-tracking of physiology may lead to better body awareness and how personality characteristics can help us predict which individuals are most likely to benefit from self-tracking of physiology.

## Figures and Tables

**Table 1:** Brief descriptions of all 20 tasks used in the experiments. The time limit of tasks was restricted to one minute, sometimes by the length of the stimulus (e.g., sound or video clips) and sometimes by sounding a bell after 1 minute and having the software automatically continue with the next step of the experiment.

Number	Category	Task description
1	Stressful	Mental arithmetic: calculate the result of 1149 minus 17, subtract 17 again from the result, etc.
2	Stressful	Read a scenario about standing in traffic, illustrated with a video.
3	Stressful	Play a computer game under time pressure, where falling cards need to be caught before they reach the bottom of the screen.
4	Stressful	Memorize a list of common English words (memory for the words is not tested afterward).
5	Stressful	Listen to a sound clip containing silence randomly interspersed with sharp bursts of noise.
6	Stressful	Count the red cars passing by in a video clip of a busy intersection.
7	Stressful	Relive a self-chosen memory that evokes frustration or anger.
8	Stressful	Make a to-do list.
9	Stressful	Watch a video clip of a throng of people getting pushed and pulled at a crowded subway station.
10	Stressful	Record a voicemail message for a job application.
11	Relaxing	Self-relaxation with eyes open.
12	Relaxing	Watch a video clip showing a quiet beach.
13	Relaxing	Play a simple computer game, where two cards with the same number need to be matched.
14	Relaxing	Watch a video of smooth, sparse traffic.
15	Relaxing	Read an article about “perfect summer weather.”
16	Relaxing	Watch a video clip showing a forest.
17	Relaxing	Read a scenario about taking a relaxing road trip, illustrated with a video.
18	Relaxing	Do a guided breathing exercise.
19	Relaxing	Relive a memory that evokes happiness or relaxation.
20	Relaxing	Listen to a sound clip containing some smooth jazz.

**Table 2:** The four statements posed after every task. Responses were given on a 7-point Likert scale ranging from “disagree completely” (1) to “agree completely” (7).

Number	Statement
1	I felt stressed during this task.
2	I felt calm during this task.
3	I felt relaxed during this task.
4	I felt tense during this task.

**Table 3:** Parameter estimates, 95% confidence intervals and significance levels for the different levels of the interactions of feedback type and personality factors on momentary self-reported stress.

Parameter	Estimate	95% CI	Sig.
Measurement-only ∗ ASI	0.05	−0.12–0.21	0.598
HR-feedback ∗ ASI	0.09	−0.12–0.29	0.406
Stress-feedback ∗ ASI	0.25	0.08–0.42	0.004^*^
Measurement-only ∗ neuroticism	0.15	0.01–0.28	0.030^*^
HR-feedback ∗ neuroticism	0.11	−0.04–0.25	0.143
Stress-feedback ∗ neuroticism	−0.05	−0.25–0.16	0.649

Dependent variable: momentary stress.

^*^Significant at *α* = 0.05.

**Table 4:** Parameter estimates, 95% confidence intervals and significance levels for the different levels of the interactions of feedback type and personality factors on retrospective self-reported stress.

Parameter	Estimate	95% CI	Sig.
Measurement-only ∗ ASI	−1.57	−4.99–1.85	0.368
HR-feedback ∗ ASI	2.79	−1.35–6.92	0.186
Stress-feedback ∗ ASI	3.58	0.15–7.01	0.041^*^
Measurement-only ∗ neuroticism	3.07	0.36–5.79	0.027^*^
HR-feedback ∗ neuroticism	2.11	−0.78–5.00	0.151
Stress-feedback ∗ neuroticism	−1.70	−5.84–2.45	0.422

Dependent variable: retrospective stress.

^*^Significant at *α* = 0.05.
